# Editorial: New advances in embryo development and embryo-endometrial interface

**DOI:** 10.3389/fendo.2026.1901952

**Published:** 2026-06-17

**Authors:** Zhenshan Yang, Julhash U. Kazi, Honglu Diao, Mingpeng Zhao

**Affiliations:** 1 Reproductive Medicine Center, Renmin Hospital, Biomedical Engineering College, Hubei University of Medicine, Shiyan, Hubei, China; 2 Hubei Clinical Research Center for Reproductive Medicine, Shiyan, Hubei, China; 3 Shiyan Key Laboratory of Reproduction and Genetics, Renmin Hospital, Hubei University of Medicine, Shiyan, Hubei, China; 4 Hubei Key Laboratory of Embryonic Stem Cell Research, Hubei University of Medicine, Shiyan, Hubei, China; 5 Division of Translational Cancer Research, Department of Laboratory Medicine, Lund University, Lund, Sweden; 6 Biomedical Research Institute, Hubei University of Medicine, Shiyan, Hubei, China; 7 Han-Peng Assisted Reproductive Technology Limited, Hong Kong, Hong Kong SAR, China

**Keywords:** decidualization, embryo development, embryo implantation, embryo-endometrial interface, endometrium

Embryo development and the embryo-endometrial interface are crucial aspects of reproductive biology, with important implications for embryo implantation, pregnancy outcomes, and maternal health (1, 2). At the developmental level, Pan et al. used spatial transcriptomics and single-nucleus RNA sequencing to construct a spatiotemporal transcriptomic atlas of the whole human embryo from Carnegie stages 12 to 23. The study resolved gene-expression dynamics across 50 developing organs and 198 substructures, identified candidate regulators of tissue identity, and uncovered previously uncharacterized gene functions in heart and brain development. This resource substantially advances understanding of human organogenesis and provides a foundation for investigating developmental disorders (3). Complementing these human developmental resources, Lin et al. generated a single-cell time-series atlas of endothelial cells across mouse embryogenesis, revealing the timing, trajectories, and molecular programs underlying organ-specific endothelial differentiation. They demonstrated that endothelial cells acquire distinct organ identities before late gestation and identified CASZ1 as a regulator of pulmonary endothelial specification, vascular growth, and epithelial–endothelial crosstalk. These findings provide important insight into the mechanisms governing endothelial heterogeneity and organ-specific vascular development (4). By analyzing a large cohort of patients with oocyte and early embryo competence defects (OECDs), Zhang et al. provided one of the most comprehensive assessments of the genetic determinants of early human reproductive failure. Their study revealed substantial genetic heterogeneity underlying defective oocyte maturation and preimplantation embryonic development, identified novel candidate regulators of developmental competence, and proposed a framework for the molecular classification of OECDs. This work advances understanding of the genetic mechanisms governing oocyte quality and early embryogenesis, with important implications for infertility diagnosis and assisted reproductive technologies (5).

A major obstacle in reproductive biology has been the limited accessibility of human implantation-stage embryos and the lack of physiologically relevant model systems. Addressing this challenge, Molè et al. developed an *in vitro* implantation platform that mimics key features of the receptive human endometrium and supports implantation and early post-implantation development of human embryos and blastoids. Their analyses revealed dynamic molecular crosstalk between embryonic and maternal cells, and perturbation experiments demonstrated that signaling between extravillous trophoblasts and endometrial stromal cells is important for trophoblast outgrowth. This model represents a significant advance for studying the cellular and molecular mechanisms that govern embryo implantation and early pregnancy establishment (6).

This Research Topic examined recent advances in embryonic development, implantation, and maternal-fetal interactions. In a large cohort of IVF patients undergoing preimplantation genetic testing for aneuploidy (PGT-A), Fan et al. showed that neither maternal thalassemia status nor euploid embryo carrier status for pathogenic thalassemia variants was independently associated with clinical pregnancy, live birth, or miscarriage. These findings support reproductive counseling for couples at genetic risk of thalassemia. Lang et al. evaluated the impact of blastocyst developmental speed on reproductive outcomes following euploid embryo transfer. They found that day-5 euploid blastocysts achieved higher implantation and live birth rates than day-6 blastocysts, particularly among women of advanced maternal age and in embryos with lower morphological quality. These findings highlight the prognostic value of developmental kinetics in embryo selection while confirming that day-6 euploid blastocysts remain a viable option for achieving successful pregnancy. Du et al. investigated the relationship between endometrial thickness and pregnancy outcomes in more than 13103 intrauterine insemination cycles (IUI). They found that greater endometrial thickness on the trigger day was associated with higher live birth rates, whereas a thin endometrium was linked to reduced reproductive success. These data support the use of endometrial thickness as a prognostic marker in IUI, while recognizing that it should not be used in isolation for clinical decision-making. Huo et al. demonstrated that the prognostic value of day-3 rapid cleavage is highly context-dependent: 11- to 16-cell embryos from younger women performed favorably, whereas rapid cleavage was associated with poorer outcomes among older patients with non-top-quality blastocysts. These results support stratified embryo assessment and individualized embryo-selection strategies. Shuai et al. evaluated the association between the estradiol-to-progesterone (E2/P) ratio prior to progesterone initiation and outcomes in hormone replacement therapy-frozen embryo transfer (HRT-FET) cycles. In this retrospective analysis of more than 25,000 cycles, clinical pregnancy and live birth rates declined progressively with E2/P ratios, suggesting that the higher pre-progesterone E2/P ratio may reflect endometrial hormonal status; prospective validation is still needed before routine clinical implementation. Tao et al. compared clinical and neonatal outcomes of day-4 versus day-5 embryo transfer in fresh IVF and intracytoplasmic sperm injection (ICSI) cycles. Overall, no significant differences were observed in pregnancy or live birth outcomes between the two transfer timings. However, in IVF cycles with single high-quality embryos, day-4 transfer showed higher clinical pregnancy rates than day-5 transfer. These findings suggest that day-4 embryo transfer is a viable alternative to day-5 transfer, with potential advantages in selected high-quality embryo cases. Geng et al. established a validated predictive model for Day-4 early blastocyst formation by Day-3 embryo parameters and showed that early blastocyst development is associated with improved embryo quality and pregnancy outcomes, supporting its value in embryo selection strategies. Zhou et al. examined whether the duration of progesterone exposure before frozen-thawed day-6 blastocyst transfer influences reproductive outcomes. Overall live birth rates were similar between day-6 and day-7 progesterone regimens. However, a significant interaction with blastocyst expansion stage was observed: early-stage blastocysts performed better with day-6 exposure, whereas late-stage blastocysts showed improved outcomes with day-7 exposure. These findings highlight the importance of individualizing progesterone timing according to blastocyst developmental stage. Liu et al. demonstrated that abnormal cleavage in early embryos is strongly influenced by patient characteristics and ovarian stimulation strategies, supporting individualized assisted reproductive technology (ART) protocols to improve early embryonic developmental patterns. Zhao et al. identified key endometrial hemodynamic and peristaltic parameters associated with repeated implantation failure and developed a predictive model integrating LASSO regression and Bayesian generalized linear modeling to assess endometrial receptivity.

**Figure 1:**
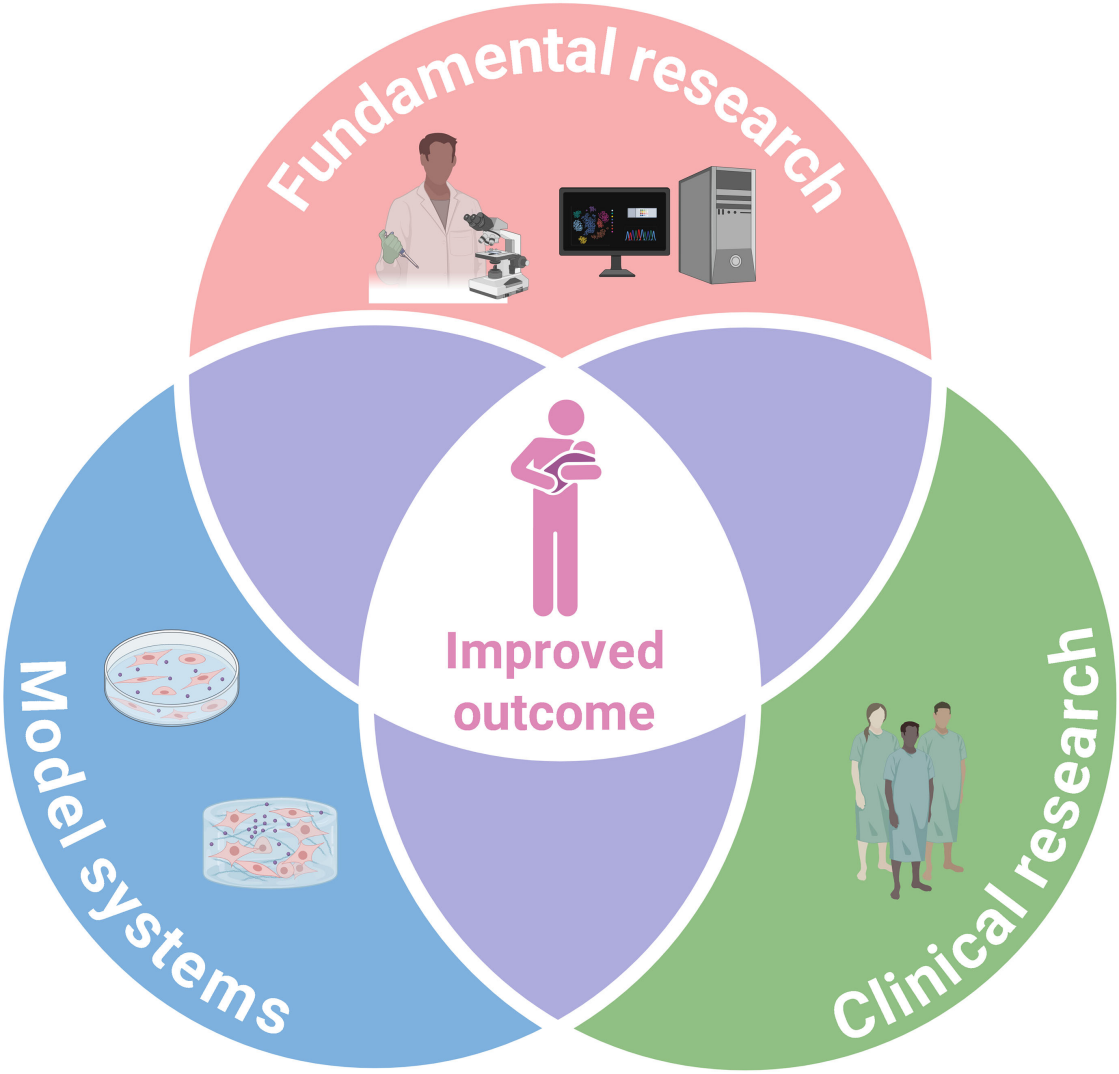
Advances in embryo development and the embryo-endometrial interface.

In conclusion, recent advances in developmental biology, reproductive genetics, and reproductive medicine have substantially expanded understanding of embryo development, implantation, and maternal–embryonic/fetal interactions. Emerging single-cell and spatial omics technologies have provided unprecedented insight into the molecular programs governing human embryogenesis, organogenesis, endothelial specialization, and endometrial receptivity, while innovative *in vitro* implantation models have enabled investigation of previously inaccessible stages of human development. At the same time, clinical studies included in this Research Topic have refined the understanding of embryo competence, embryo selection strategies, endometrial factors, and hormonal regulation, offering valuable evidence to improve assisted reproductive technologies and reproductive outcomes (**Figure 1**). Continued interdisciplinary efforts combining multi-omics technologies, physiologically relevant modeling, and large-scale clinical investigations will further deepen understanding of early human development and facilitate personalized reproductive medicine.
